# A Report upon Some Late Statements in Therapeutics

**Published:** 1844-06-15

**Authors:** W. Hughes Willshire


### A REPORT UPON SOME LATE STATEMENTS IN THERAPEUTICS.

BY W. HUGHES WILLSHIRE, M. D., M. B. S., ETC.

*Impetigo*—When occurring in infants during the first dentition, Erichsen, Plenck, Granville, Billard, Biett, and others, doubt the propriety of interfering, and driving in the eruption suddenly. According to Erichsen, in the acute stage in older persons, all specific remedies are perfectly useless. In the more chronic stages, Erichsen has found a lotion of the sulphuret of potash, with sulphurous waters taken internally, the best mode of treatment. Cazenave and Schedal, although advising a lotion of sulphuret of potash, carbonate of potash or soda, and water, state that the preparations of sulphur have been too generally recommended, and that their indiscriminate employment, especially in the earlier stages, is often decidedly injurious. In some instances, Erichsen uses an ointment of the nitrate of mercury, or else of the peroxide; and if the itching is very troublesome, a lotion of the oxide of zinc, with a little prussic acid, is advisable.

Rayer approves of sulphurous lotions, and ointments of the nitrate of mercury; and Dr. Thomson has seen the best results from the use of the latter. A combination of the acetate of lead and prussic acid, or the application of blisters to the part, have also been recommended. The internal remedies of late spoken well of, are: arsenic, sulphur, nitric acid, and the bichloride of mercury.

*Lichen.*—According to Duvergie, tepid baths are useful at the beginning; alcaline or sulphurous ones are too irritating. Vapour or Russian baths, or those containing deuto-chloride of mercury, are the most efficacious. Alone, however, they are insufficient, and internal remedies must be added, and the best is the tincture of cantharides. A child seven years of age may take eight minims every evening.

*Prurigo.*—In P. senilis, Mr. Wilson has used iodine locally with good effect. M. Biett relies upon the ioduret of sulphur (gr. xx-xxx+℥j); and creosote, sulphur, and the oxide of zinc, are spoken well of by others. The author has found the nitrate of silver to be the best application in P. pudend. muliebris.

*Lepra and Psoriasis.*—The following things have of late been recommended by Biett:—Warm sulphurous, or salt water baths; towards the decline an ointment of the ioduret of sulphur; by Gibert an ointment of soot and potash; and this Mr. Wilson has also recommended; by Lemarq, an ointment of naphthaline; by Plumbe, a compound of calomel, nitrate of mercury, and the acetate of lead. Internally, Biett recommends purgatives, tincture of cantharides, and the preparations of arsenic. Cazenave and Schedal speak well of the cantharides in particular forms of these maladies. Dr. Thomson prefers the iodide of arsenic to Fowler's solution; and more recently, Donovan has introduced a combination of arsenic, iodine, and mercury. The author has very lately employed the binoxide of manganese, with temporary relief. Biett states that he has not found the decoction of dulcamara of much use; tar and pitch have invariably failed with him; and sarsaparilla, mezereon, and white hellebore are very uncertain.

*Rupia.*—In the form *R. escharotica*, Dr. Stokes has advised an ointment of the *scrophularia nodosa* along with the internal administration of yeast. This affection, which occurs in debilitated, ill-fed, dirty children, the author has repeatedly treated beneficially by the chlorate of potash and quinine. A lotion of the sulphate of zinc may at the same time be employed.

*Molluscum.*—Gibert states the several species comprehended under this title, to be generally incurable.

Duvergie has treated the *sycosis menti* and *acne rosacea*, of Willan, by the sulphate of iron. It is used in solution, either by bathing the part affected, or by applying linen dipped in it, or by sprinkling the ulcerated parts of the *sycosis* with a mixture of charcoal and sulphate of iron. The following remarks were made by an anonymous writer, in Johnson's Review:—"With respect to the sulphate of iron, as an external application, we cannot believe that it possesses any curative virtues above those of the sulphate of zinc, or of the sulphate of copper, that are in daily use. The white vitriol is our favourite, and the best way of applying it is by dipping rags of soft linen in a tepid solution of the salt, and covering these with a piece of oil-skin." In some cases, a little hydrocyanic acid may be well added to the solution. The liquor potassæ, given in doses of from 15 to 20 drops, three times a-day, is an admirable remedy in many cases of inveterate skin disease. According to our observations, it is far more efficacious, and perhaps, too, less injurious than the potash in combination with iodine. It may be given in milk, beer, decoction of sarsaparilla, &c.

In a case of *elephantiasis Barbadensis* cured by

Cazenave, the following plan was adopted:—The patient took a strong decoction of guaiacum and mezereon, used vapour baths, rubbed the leg with ung. hyd. potassæ, and had it bandaged from the toes upwards; absolute rest in bed was also enjoined. It has been remarked that the practice adopted was extremely judicious. The guaiacum and mezereon often exert a very marked influence on the cutaneous circulation, stimulating the exhalants, and invigorating the capillary vessels; hence they are often excellent remedies in many cases of chronic skin disease, rheumatism, &c. The natives of the Sandwich Islands suffer from a species of leprosy, which they call *craw-craw;* to cure it, they have recourse to the *cava-root*, a very powerful narcotic and alterative.

*Lupus.*—Lisfranc recommends the liquid acid proto-nitrate of mercury to be applied when the local vascular action has been subdued or moderated. The part is touched lightly with the caustic; the object is not to disorganize it wholly, but rather to change its action in some mysterious way, designated by M. Lisfranc a modification of the vitality of the tissues. It is said that the great fault of this remedy is, the long persistence of severe pain after its application.

Mr. James treats *noli me tangere* with the most complete success by a compound of the chloride of zinc (1 part), and sulphate of lime (3 parts). This is moistened and applied on lint over a small portion of the diseased surface; after about five days it falls off, exposing a healthy-looking sore, which readily heals on the use of a simple dressing.

In *cancer of the uterus* according to Lever, arsenic has, unquestionably, often considerable power in lessening severe pains, if not in controlling the morbid action. He reports, also, favourably of iodine, at least before ulceration has taken place.

In *cancer of the nose*, three-fourths of which organ had been removed by Lisfranc, on account of the ravages of the disease, and it having re-appeared, the patient is said to have been cured by the use of the iodide of potassium internally, and simple ointment externally.

Mr. Tuson has employed, with temporary relief to the patient, the chlorides of zinc and lead. Of the former he remarks that it cannot be doubted that its external use is beneficial in healing open cancer—a solution of ʒj. to a pint may be employed; other preparations of chlorine are also sometimes of use: chloride of lime, chloride of lead, ter-chloride of carbon, per chloride of copper, &c., and a case has been described of a lady completely healing a large open cancer by the application of the chloride of sodium. The chloride of lead has been very extensively used in many cases of disease of the breast and uterus, in the form both of lotion and ointment, with much success; it appears to act upon the nerves, paralysing them and thus producing ease. A drachm of the chloride of lead to a pint of water may be used as a lotion, and a draught containing ten grains of the chloride of potassium may be taken three times a day.

According to Brodie, there is a *disease of the tongue* which is like a malignant one, in many respects; it is often mistaken for cancer, though it is of a different nature. In some instances, the disease may be relieved by a course of sarsaparilla, with small doses of the bichloride of mercury; but the remedy best adapted for these cases is a solution of arsenic. Give the patient five minims three times a day, in a draught, gradually increasing the dose to ten minims. In some instances, however, it will take a long time to get the tongue quite well, and then you may give the former remedy at one time, the arsenic at another. You cannot give either of these remedies for a very long time continuously; but it is astonishing what bad tongues will get well under these modes of treatment.

In *corroding ulcer of the uterus*, as distinguished from carcinoma, Dr. Lever is in the habit of applying the nitrate of silver, either in substance, or in solution. Tepid milk and water, containing syrup of poppies, or liquor opii, may also be injected.

On the *treatment of syphilis*, Sir Alexander Crichton is convinced that Fricke's plan of treatment—cleanliness, low diet, and laxative medicine—will not succeed in private practice, because patients will not submit to the restrictions imposed. Whilst Cruvelhier, Ricord and Chassaguy, regard ptyalism as the most grave accident accompanying the use of mercury in syphilis, Dr. Muynell affirms, that a person cannot be pronounced as cured unless he has been subjected to ptyalism. According to Brodie, the best way of using mercury, when the disease is severe, is by inunction. It has its inconveniences; but cases will occur in which you may patch up the disease by giving mercury internally, but it will return again and again, and then you will cure it at last by a good course of mercurial inunction. Of syphilitic children, Sir Benjamin states that very few recover to whom this drug has been given internally; but if the mercurial ointment be spread on a flannel roller, and the latter applied round the knee, not only no untoward effects will ensue, but the child will be cured by it. With respect to the effect of mercury upon the system generally, you cannot depend upon its effects in syphilis, unless the gums be made rather sore, and there be some degree of ptyalism.

Mr. Markwick reports a case of *sloughing venereal ulcer* cured by the application of powdered rhubarb.

*Strangulated Hernia.*—Lyell records a successful case. Within three hours, the patient had three grains of opium and four and a half of hydro-chlorate of morphia; and the rule which the above writer would recommend is to employ the morphia in half-grain hourly or half-hourly doses, till the patient is fairly narcotised.

Walker mentions the case of a patient with *strangulated scrotal hernia*, which became reduced after two grains of opium were taken, every fifteen minutes, for forty-five minutes.

Tapcot mentions that an enema made with ℈xv. of tobacco, administered to a man 56 years of age, labouring under strangulated hernia, produced symptoms of narcotism in a quarter of an hour, the poor fellow dying ten minutes afterwards.

When inflammatory symptoms follow reduction, Lisfranc considers the internal application of mercurial ointment to be highly efficacious; according to the formula of M. Serres d'Uzes, two pounds of the ointment are used in the course of 48 hours, spread on the lateral and anterior parts of the abdomen, laid on layer by layer every two hours, until the whole dose is expended. M. Lisfranc has thus used sixteen pounds in eight days.

In *bed sores* nitrate of silver is recommended by Jackson. The form which he uses is in the proportion of ten grains to the ounce, applied by means of a camel-hair brush over every part exhibiting the slightest appearance of inflammation, two or three times a day, until the skin has become blackened; afterwards only occasionally.

A correspondent of the French Academy recommends *nævi materni* to be inoculated by a lancet charged with croton oil; each of the punctures becomes the seat of a boil.—*Lond. Med. Times.*

